# Direct intra-abdominal pressure monitoring via piezoresistive pressure measurement: a technical note

**DOI:** 10.1186/1471-2482-9-5

**Published:** 2009-04-21

**Authors:** Jens Otto, Daniel Kaemmer, Marcel Binnebösel, Marc Jansen, Rolf Dembinski, Volker Schumpelick, Alexander Schachtrupp

**Affiliations:** 1 Department of Surgery, University Hospital RWTH Aachen, Aachen, Germany; 2 Department of Surgical Intensive Care Medicine, University Hospital of the RWTH Aachen, Aachen, Germany

## Abstract

**Background:**

Piezoresistive pressure measurement technique (PRM) has previously been applied for direct IAP measurement in a porcine model using two different devices. Aim of this clinical study was to assess both devices regarding complications, reliability and agreement with IVP in patients undergoing elective abdominal surgery.

**Methods:**

A prospective cohort study was performed in 20 patients randomly scheduled to receive PRM either by a Coach^®^-probe or an Accurate++^®^-probe (both MIPM, Mammendorf, Germany). Probes were placed on the greater omentum and passed through the abdominal wall paralleling routine drainages. PRM was compared with IVP measurement by t-testing and by calculating mean difference as well as limits of agreement (LA).

**Results:**

There were no probe related complications. Due to technical limitations, data could be collected in 3/10 patients with Coach^® ^and in 7/10 patients with Accurate++^®^. Analysis was carried out only for Accurate++^®^. Mean values did not differ to mean IVP values. Mean difference to IVP was 0.1 ± 2.8 mmHg (LA: -5.5 to 5.6 mmHg).

**Conclusion:**

Direct IAP measurement was clinically uneventful. Although results of Accurate++^® ^were comparable to IVP, the device might be too fragile for IAP measurements in the clinical setting. Local ethical committee trial registration: EK2024

## Background

Intra-abdominal hypertension (IAH) and abdominal compartment syndrome (ACS) have been observed to occur in any patient population needing intensive care with an incidence rate of 37% and 7% respectively [1,2].

Intra-abdominal pressure (IAP) measurement has been recommended in patients at risk to develop IAH and ACS [3]. The gold standard for intermittent IAP measurement is the intra-vesicular pressure measurement (IVP) [3]. This measurement principle is widely accepted in the clinical regard [4-6]. But it has inherent problems with regard to intrinsic bladder wall tension, reference level [7], body position, discontinuity and indirectness [8].

In a previously described porcine model two different devices capable of automatic continuous piezoresistive pressure reading measurement (PRM) were used for direct IAP measurement. Both, a probe to be connected to a handheld reading device and the other with the ability to perform a reset to the atmospheric pressure showed a high precision and a good agreement with bladder pressure measurement[9,10]. Although direct intraabdominal pressure measurement is routinely used to validate indirect methods [11-14] it has not yet been systematically evaluated whether these PRM techniques can be performed safely and reliably in the postoperative monitoring of patients.

Aim of the underlying study was to evaluate PRM for direct measurement with regard to feasibility, complications and agreement with bladder pressure measurement in patients undergoing elective abdominal surgery.

## Methods

With approval of the local ethical committee (document-nr. EK-2024) a prospective cohort study was performed between January and August 2003 at the surgical intensive care unit (ICU) of the Department of Surgery, University Hospital of the RWTH Aachen, Germany. The study was conducted in accordance with the study protocol, the Declaration of Helsinki and applicable regulatory requirements.

Study participants were recruited from patients scheduled for elective abdominal surgery after informed and written consent was obtained on the day before surgery was performed. Study participants received colonic resection (n = 7), oesophageal resection (n = 4), pancreaticoduodenectomy (n = 2), gastrectomy (n = 4), liver resection (n = 2) and incisional hernia repair (n = 1). Patients were included if an abdominal drainage was placed and if postoperative ICU surveillance as well as placement of a Foley-catheter was deemed necessary due to the standard surgical procedure (not for study reasons).

Excluded were patients with an age < 18 years, coagulation dysfunction, intraabdominal inflammation, liver insufficiency (Child-Pugh-stage B or C), renal failure with necessity for dialysis and inclusion in other studies.

### Piezoresistive Measurement of IAP

Measurement of IAP via piezoresistive pressure measurement was done with two different probes implanted in 20 patients in randomised order.

In 10 patients the Coach^®^-system (CPRM, MIPM, Mammendorf, Germany) was used. This system has been used for measurement of the pressure within the intramuscular compartment [15]. It consists of a unicrystalline piezo-semiconductor on the tip of a polyurethane coated catheter (outer diameter 1.35 mm) which is connected to a hand-held reading device. At the moment the probe is connected to the reading device, the pressure display is automatically set to zero on the basis of the surrounding pressure. Accordingly, a correct reset to zero cannot be performed if the probe is exposed to a pressure being different from the atmospheric pressure, e.g. in-situ. Setting to zero was therefore performed only once in the beginning of the investigation during the intraoperative placement. Pressure readings are displayed in mmHg. The probe is reusable after resterilization (120°C steaming for 20 minutes).

In another group of 10 patients the Accurate++^®^-probe (APRM, MIPM, Mammendorf, Germany,) was used. This probe has been designed to measure the intracerebral pressure. The pressure sensor of this probe is covered by a membrane and the outer diameter is 5 mm. The probe can be exposed to the atmospheric pressure allowing a reset to zero in-situ. APRM can be connected to every standard ICU-monitor without a special reading device. Pressure readings are displayed in mmHg. The probe is reusable after resterilization (120°C steaming for 20 minutes). After use in patients, both types of probes were mechanically cleansed and the function tested. If functioning correctly, they were sent to sterilization. Otherwise, they were sent to the manufacturer for repair.

### Intravesicular pressure measurement

For hydrostatic intravesicular pressure (IVP) measurement in both groups, the tubing system, Foley catheter, and bladder were firstly flushed with 50 ml sterile saline. This fluid was completely drained leaving no air in situ before another 50 ml saline was injected serving as measurement volume. Using a standpipe, pressure readings were obtained at the end of the expiration. The level of the symphysis always served as reference and readings in cmH_2_O were converted into mmHg by multiplication with 0.74.

### Measurement protocol

For direct IAP measurement, CRPM or APRM probes were placed on the greater omentum in midline position cranial of the umbilicus at the end of the operation. Catheters were then passed through the abdominal wall paralleling the routinely used drainages (Easy Flow^®^) and were fixed to the skin with a suture. The probes were connected to the reading device (CPRM) or to the hemodynamic monitor (APRM) and the surgeon was asked to gently squeeze the probe with his fingers in order to test overall function of catheter and monitor. Afterwards, the abdomen was closed.

IVP measurements were done every 8 hours according to a protocol. PRM measurement was performed continuously. Pairwise readings of PRM and IVP were used for further analysis.

The intraabdominal measurement probes were withdrawn whenever the urinary catheter was removed, patients left the intensive care unit, or after 5 days of PRM measurement. They were also withdrawn if malfunction occurred. This was given if no reading was displayed or if readings did not change with squeezing, breathing or due to a gentle pressure placed manually on the abdomen. When CPRM was found to be disconnected malfunction was also assumed and the probe was withdrawn. The overall incidence of malfunction was recorded.

Patients were physically examined during IVP measurement and assessed for probe malfunction as well as for catheter related erosion and infection of adjacent tissue.

### Statistical analysis

Results are presented as mean ± SD. To compare readings derived from IVP and PRM Student's t-test was applied. Moreover, the mean difference and limits of agreement (mean difference ± 1.96 SD) were calculated according to the method of Bland and Altman [16].

## Results

Patients had a mean age of 57.1 years. The mean body weight was 75.4 kg with a mean body-mass-index (BMI) of 24.4.

In one patient scheduled to receive CPRM, no signal could be retrieved intra-operatively due to a break in the conductive path. In six from the remaining 9 patients measurements had to be aborted ahead of schedule because of disconnection (2 patients) or offset-failure due to a fibrin-encrustration (4 patients). Thus, only 3 of 10 patients were examined according to protocol leading to an overall measurement rate of 0.3.

In two patients scheduled for APRM readings did not change with application of manual pressure. In both cases, probes were not inserted. However, probes turned out to function properly afterwards as confirmed by the manufacturer. In one of the 8 remaining patients an offset-failure was observed. Accordingly, the overall measurement rate was 0.7.

Postoperative course of all patients was uneventful. There were no signs of probe related organ lesion or surgical site infection. Withdrawal of the measurement probe at the end of the measurement period could be done uneventfully in all patients.

**Figure 1:**
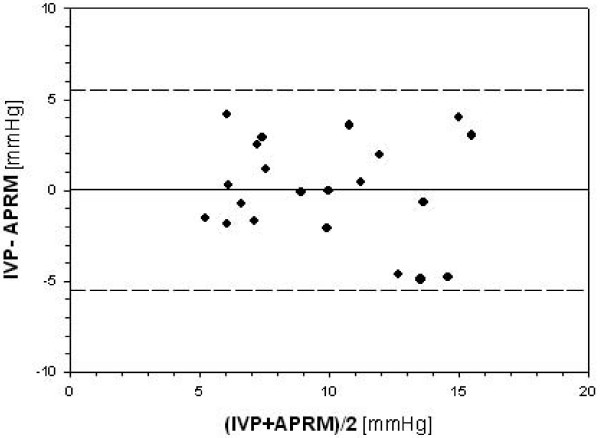
**Pairwise measurements (n = 21) of intravesicular pressure and intraabdominal pressure measured directly using a piezoresistive probe (APRM)**. The probe was place on the greater omentum in 7 patients undergoing elective abdominal surgery. Difference vs. mean value according to Bland and Altman [16].

Comparing CPRM with IVP only 5 pair wise measurements could be recorded. Because of the scarcity of data, no further analysis was carried out. Comparing APRM with IVP 21 pairwise measurements were recorded. Mean APRM reading was of 9.8 ± 3.7 mmHg while mean IVP reading was 9.9 ± 4.4 mmHg (p = 0.96). The mean difference between IVP and Accurate^®^-probe was 0.1 ± 2.8 mmHg. Limits of agreement were -5.5 mmHg to 5.6 mmHg (figure 1).

## Discussion

Direct measurement of the intraabdominal pressure has been regarded to be invasive [17]. In contrast, intraperitoneal measurement has also been considered to be needed to fully address the accuracy of IAP measurement in clinical practice [18]. Currently, direct measurement of IAP is routinely applied for validation of indirect techniques [11-14].

In the underlying study, placement of an intraabdominal measurement probe in patients undergoing elective abdominal surgery did not lead to adverse effects. This is in accordance to Brooks and co-workers, who recently evaluated a device for a direct and continuous assessment of IAP and reported no complications [19].

The use of CPRM was associated with frequent technical difficulties during the study. Disconnection of this measurement system occurred in two patients. As a reset to the atmospheric pressure (zeroing) cannot be performed reliably in vitro, readings were not credible. Consequently, probes were withdrawn. Furthermore, malfunction was found in five patients.

APRM in contrast was applicable in 7 of 10 patients and an offset-failure could be noticed in one case only. The other two malfunctions remain unclarified but could result from faulty operating of these sensitive devices. Exploring the occurrence of technical difficulties we could not assess a learning curve contamination bias.

While pair wise measurements in CPRM were too sparse for a reliable analysis, only APRM could be used for altogether 21 recordings. Mean difference to IVP was 0.05 mmHg with limits of agreement ranging from -5.5 mmHg to 5.6. To our knowledge, this kind of piezoresistive pressure measurement probe has not been used for assessment of IAP yet.

The consensus conference definitions and recommendations on IAH and ACS stated that a new IAP measurement technique should have a mean difference from -1 to 1 mmHg and limits of agreement within 4 mmHg [18]. In this concern, agreement of APRM with IVP was only moderate and might be explained by the fact that measurements were performed in two different compartments as already pointed out in other clinical investigations [20,21].

Regarding the limited amount of measurements and a measurement rate of 0.7, it appears that APRM is basically capable to measure the IAP reliably but is probably too fragile for the setting of the underlying study. To improve the setting the probes may be put into the rectus abdominis muscle space in order to avoid fibrin-encrustration like Meier et al. explored in an experimental model [22]. Furthermore it remains to be tested how the systems may work once exposed to very high pressures inside the abdominal cavity.

The measurement volume has been described to falsely induce intrinsic pressure when exceeding 25 ml [23]. Malbrain could prove that instilling over 50 ml of saline into the bladder may overestimate actual IAP. He state that 25 ml may be enough to prime the bladder for estimation of IAP [24-26]. Kimball recently published a study in which bladder pressure measurement in critically ill patients using 50 ml displayed high reproducibility and reliability [27]. Consequently, the 50 ml used as measurement volume for IVP in the patients of the underlying study appear to be appropriate.

The facility of continous IAP measurement using piezoresistive pressure monitoring may lead to a more frequent execution in the ICU. In this context we have to complement that there are several indirect techniques (gastric, direct abdominal, inferior vena cava, and urinary bladder) of continous IAP monitoring [17].

## Conclusion

Intraperitoneal measurement of IAP was performed in 17 patients after elective abdominal without adverse effects. Regarding piezoresistive measurement probe with the possibility for in-vitro reset to zero, agreement with standard IVP was acceptable. However, the device might be too fragile for routine IAP measurements in the clinical setting.

## Competing interests

A.S. is Scientific Manager for B. Braun Melsungen, Germany which does not manufacture a commercially available kit for intra-abdominal pressure monitoring. The remaining authors have no financial involvement with any organization or entity with a financial interest in or in financial competition with the subject matter or materials discussed in the manuscript.

## Authors' contributions

J.O. and A.S. have made substantial contributions to conception and design. D.K. and M.J. have been involved in revising the manuscript critically for important intellectual content. M.B. and R.D. have made substantial contributions to acquisation of data. V.S. has been involved in analysis and interpretation of data and has given final approval of the version to be published.

## Pre-publication history

The pre-publication history for this paper can be accessed here:


